# Mortality Risk among Children Admitted in a Large-Scale Nutritional Program in Niger, 2006

**DOI:** 10.1371/journal.pone.0004313

**Published:** 2009-01-29

**Authors:** Nael Lapidus, Andrea Minetti, Ali Djibo, Philippe J. Guerin, Sarah Hustache, Valérie Gaboulaud, Rebecca F. Grais

**Affiliations:** 1 Epicentre, Paris, France; 2 Ministry of Health, Niamey, Niger; National Institute for Public Health and the Environment, Netherlands

## Abstract

**Background:**

In 2006, the Médecins sans Frontières nutritional program in the region of Maradi (Niger) included 68,001 children 6–59 months of age with either moderate or severe malnutrition, according to the NCHS reference (weight-for-height<80% of the NCHS median, and/or mid-upper arm circumference<110 mm for children taller than 65 cm and/or presence of bipedal edema). Our objective was to identify baseline risk factors for death among children diagnosed with severe malnutrition using the newly introduced WHO growth standards. As the release of WHO growth standards changed the definition of severe malnutrition, which now includes many children formerly identified as moderately malnourished with the NCHS reference, studying this new category of children is crucial.

**Methodology:**

Program monitoring data were collected from the medical records of all children admitted in the program. Data included age, sex, height, weight, MUAC, clinical signs on admission including edema, and type of discharge (recovery, death, and default/loss to follow up). Additional data included results of a malaria rapid diagnostic test due to *Plasmodium falciparum* (Paracheck®) and whether the child was a resident of the region of Maradi or came from bordering Nigeria to seek treatment. Multivariate logistic regression was performed on a subset of 27,687 children meeting the new WHO growth standards criteria for severe malnutrition (weight-for-height<−3 Z score, mid-upper arm circumference<110 mm for children taller than 65 cm or presence of bipedal edema). We explored two different models: one with only basic anthropometric data and a second model that included perfunctory clinical signs.

**Principal Findings:**

In the first model including only weight, height, sex and presence of edema, the risk factors retained were the weight/height^1.84^ ratio (OR: 5,774; 95% CI: [2,284; 14,594]) and presence of edema (7.51 [5.12; 11.0]). A second model, taking into account supplementary data from perfunctory clinical examination, identified other risk factors for death: apathy (9.71 [6.92; 13.6]), pallor (2.25 [1.25; 4.05]), anorexia (1.89 [1.35; 2.66]), fever>38.5°C (1.83 [1.25; 2.69]), and age below 1 year (1.42 [1.01; 1.99]).

**Conclusions:**

Although clinicians will continue to perform screening using clinical signs and anthropometry, these risk indicators may provide additional criteria for the assessment of absolute and relative risk of death. Better appraisal of the child's risk of death may help orientate the child towards either hospitalization or ambulatory care. As the transition from the NCHS growth reference to the WHO standards will increase the number of children classified as severely malnourished, further studies should explore means to identify children at highest risk of death within this group using simple and standardized indicators.

## Introduction

Several Sub-Saharan countries experience chronic food insecurity. The intake of food, in terms of both quality and quantity, is insufficient to meet the nutritional needs of children especially in their first years of life. This combined with other diseases such as malaria, measles, diarrhea or pneumopathies leads to high mortality in children under five. Niger is particularly affected by chronic food insecurity, marked each year with a “lean season” or “hunger gap”, period when the previous year's stocks have run out but the new crop is not yet ready for harvest. This period usually lasts from three to six months. The child mortality risk in Niger, defined as risk of death before age 5, is one of the highest in the world (198 per 1000 [1]).

In 2005, Niger experienced an unusual nutritional crisis, a consequence of chronic food insecurity aggravated by drought and speculation on food prices, which particularly affected rural areas. One of the most affected regions was the department of Maradi, in south Niger where up to one in five children were estimated to suffer from global (i.e. either moderate or severe) acute malnutrition [2]. In collaboration with the Ministry of Health, the medical aid agency Médecins sans Frontières (MSF) treated over 40,000 severely wasted children in the country during 2005.

Although severely malnourished children have a higher relative risk of mortality, the much larger numbers of moderately malnourished children leads to a higher population-attributable risk for mortality in this group [3], [4]. Therefore, in 2006 MSF expanded its nutritional intervention to target children at an earlier stage. Children aged 6 to 59 months, presenting with either moderate or severe wasting, according to the US National Center for Health Statistics (NCHS) reference, were admitted to the program.

**Figure 1:**
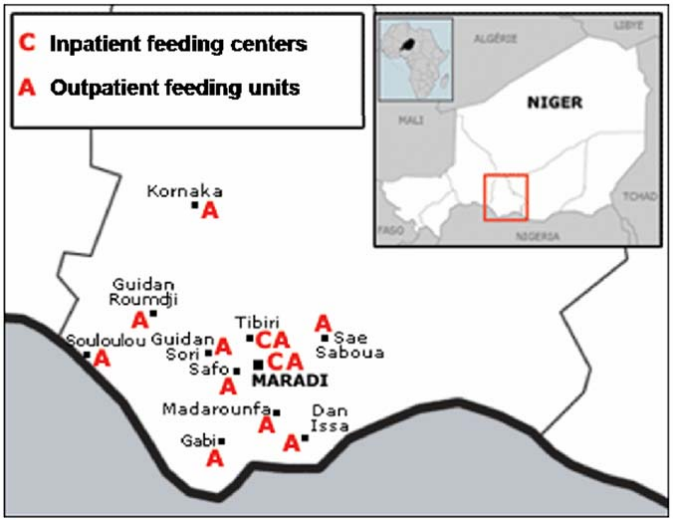
Map of locations of MSF inpatient centers and outpatient units in Maradi, Niger 2006.

In 2006, MSF operated 11 outpatient centers attached to integrated health centers, along with 2 inpatient referral centers (figure 1). The large-scale outpatient care was based on weekly follow up and treatment with Ready-to-Use-Therapeutic-Foods (Plumpy'nut®, Malaunay, France) delivered by mobile teams comprised of nurses. Inpatient treatment of complicated cases (e.g., presenting with infection, severe dehydration, consciousness disorders) relied on a therapeutic diet or RUTF, in addition to treatment for complications. Free consultation and treatment were also accessible for all children under-five in the area.

Information on mortality risk is critical for the improvement of guidelines for quality care of malnourished children and to identify areas where future research and reflection are needed. A score of individual mortality risk could be proposed to identify children at highest risk of death for whom more intensive care may be needed, in settings where thorough medical examination is not possible for all children, as in many community-based programs for the treatment of malnutrition.

In 2006, the World Health Organization (WHO) introduced new growth standards, which replace the NCHS reference as anthropometric criteria for admission to nutritional programs [5]. With these new standards, regarding weight-for-height (WH), many children now considered severely malnourished were identified formerly as moderately malnourished by the NCHS reference [6], [7]. Thus, there is a need to reassess risk ratios of death in children defined as severely wasted using these new growth standards instead of the former NCHS reference.

Prudhon *et al* used data on 3,858 severely malnourished children admitted to feeding centers during emergency operations to determine a simple model using height, weight and presence of edema on admission to predict an individual probability of death using the NCHS reference [8]. Principal factors for mortality in children hospitalized with severe malnutrition (for example, acute bacterial infections, electrolyte imbalance, and micronutrient deficiencies [9]–[12]) have been identified only for severe wasting as defined by the NCHS reference. To our knowledge, there has not been a study aimed at identifying risk factors for mortality for severely malnourished children according to the new 2006 WHO standards.

The MSF program represents a large cohort providing a unique opportunity to study the risk factors of death in acutely malnourished children. Here, we provide an overview of the nutritional program and then report on our analysis of risk factors for death among children admitted to the MSF program in 2006.

## Methods

### Study Site

The district of Maradi is located along the southern border of Niger with Nigeria. The region has the highest rates of acute malnutrition in the country; highest in both of the districts where MSF concentrates most of its nutritional and health activities. The prevalence of global acute malnutrition in Maradi was estimated to be 11.6% between January and May 2006 and 16.0% in October 2006. The prevalence of severe acute malnutrition was estimated to be 1.0% between January and May and 2.3% in October [1], [13].

### Study Population

The study population included all children admitted to the MSF nutritional program in Maradi, Niger, during 2006. Measurements were taken in accordance with WHO standard techniques and compared with the NCHS reference population [14], [15].

At admission, children were screened for malnutrition using weight, height (or length for children less than 85 cm) and presence of edema. Children were eligible for program admission when meeting at least one of the following criteria: weight-for-height less than 80% of the median (NCHS), mid-upper arm circumference (MUAC) less than 110 mm (if height above 65 cm) or bipedal edema. Discharge occurred at WH greater than 80% of the median at two consecutive weighings and at WH>85% of the median on one weighing after early November.

Acute malnutrition cases were classified as complicated or non-complicated [16]. Complicated acute malnutrition was defined as either moderate or severe acute malnutrition accompanied by anorexia and/or kwashiorkor with major edema and/or severe pathology, i.e. illness requiring specific care in a hospitalized structure. Complicated cases were admitted to one of the two inpatient centers for treatment and stabilization. Non-complicated cases – either severe or moderate – were managed by the outpatient units and were referred to inpatient centers only if they developed complications during the course of their treatment or were not responding to treatment. Non-complicated moderately and severely malnourished children were treated with the same medical and dietary protocols, except that the severely malnourished had systematic antibiotic treatment at admission. RUTF consisted of two daily packets of Plumpy'nut® (1,000 kcal/day).

All children were assessed initially by trained nurses running the outpatient program, who established whether the child could be managed as outpatient or inpatient. Only the latter were examined by a physician. This approach, relying on trained nurses using risk factors of death in malnourished children, allowed for the coverage of a large number of children, who could not possibly be examined by physicians at first-line admission.

The nurses in charge of the triage used the following clearly defined clinical criteria. Anorexia was determined by whether a child refused to eat RUTF. Children were given an open packet of Plumpy'nut® to eat during admission and were observed for approximately 10 minutes while administrative data were collected from the mother. If the child did not eat during this period, they were considered anorexic; Plumpy'nut® is known and liked by children in Maradi. Mothers were asked about recent (24 hours) or ongoing vomiting and diarrhea. Temperature above 38.5°C was considered febrile. Pallor was determined by examining the inner eyelids. Apathy was defined as a poor response to stimulation.

### Inclusion in the analysis

**Figure 2:**
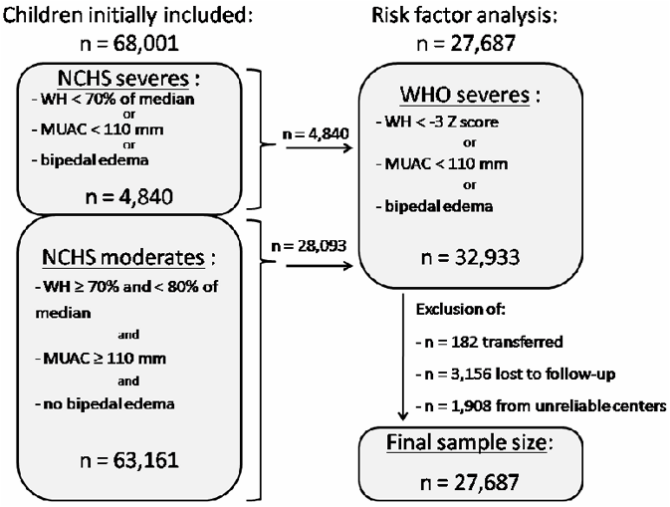
Flow diagram of observations retained in the analysis.

Although the management protocol in 2006 focused on complicated versus non-complicated cases, data are presented here in terms of anthropometrical severity, to facilitate analysis and for the sake of clarity. Severe malnutrition was defined as WH less than 70% of the median (NCHS), MUAC less than 110 mm (for children taller than 65 cm) or presence of bipedal edema (“NCHS severes” on the flow diagram, figure 2).

For the risk factors analysis, we excluded children that had missing weight, height, age or gender information. Children with missing information on edema status (n = 10,724) were considered not to have edema as clinicians were asked to note only when edema was present. We also excluded children transferred to other organizations or facilities (n = 182), as the reasons for transfer were not documented; children lost to follow up or with missing final status (n = 3,156); and, children from 3 outpatient centers that were considered to produce unreliable data (n = 1,908). In order to identify risk factors on a population similar to those likely to be found in nutritional programs in the future, we restricted our analyses to children meeting the definition of severe malnutrition according to the WHO growth standards (“WHO severes” on the flow diagram, figure 2); weight-for-height less than −3 Z score, MUAC less than 110 mm (when height above 65 cm) or bipedal edema (n = 32,933).

### Data Analysis

Information was extracted from medical records of discharged children using standardized data collection forms and entered in a program monitoring database on a weekly basis. Data collected included age, sex, height, weight, MUAC, edema status and clinical signs on admission, and type of discharge (recovery, death, and default/loss to follow up). Additional data collected included results of a malaria rapid diagnostic test due to *Plasmodium falciparum* (Paracheck®) and whether the child was a resident of the region of Maradi or came from bordering Nigeria to seek treatment.

We chose to examine two different multivariate logistic regression models to estimate the contribution of each potential risk factor for death. A first regression took into account only height, weight, gender and presence of edema. These covariates are those routinely collected at admission and can be assessed by less trained medical staff. Variables of clinical importance, requiring more training, were added in a second regression analysis. Independent variables that persistently showed non-significant relationships with death were excluded from the final models. We included interaction terms one at a time in the near final models and explored interactions within the multivariate model. Both step-wise (forward and backward selection) and completely saturated models were tested. Data was stratified by age and gender and a seasonal forcing term included in the model to account for the seasonal nature of malaria and other diseases. To account for potential differences between treatment units, all analyses were performed with multilevel models. During modeling, we retained variables with statistical significance of p<0.05, using odds ratio, with minimization of the AIC criterion. We derived and present two formulae, one for each regression, to estimate the individual risk of death. These formulae can be considered as an update to the Prudhon index [6] adapted to the new WHO criteria of severe acute malnutrition.

All analyses were conducted with R version 2.6.0 (R Development Core Team; R Foundation for Statistical Computing, [http://www.R-project.org]). The risk factor analysis was performed with the R package lme4: Linear mixed-effects models using S4 classes version 0.999375-27.

### Ethical considerations

We used routine monitoring data from the MSF nutritional program, which was conducted in coordination with the Ministry of Health via a memorandum of understanding, which is the usual procedure for NGOs operating in these contexts. No supplementary interventions were conducted for the analysis presented here. All electronic data were entered anonymously and identifiers were coded. No ethnic or identifying information was entered.

## Results

### Program Overview

In 2006, 68,001 children aged 6–59 months were admitted to the MSF program. Of these, 92.9% (n = 63,161) presented with moderate acute malnutrition and 7.1% with severe malnutrition (n = 4,840). Table 1 provides the mean and the median weight, height or length, and age for children entering the program. Of 60,755 children with documented MUAC, 2,008 (3.3%) had a MUAC<110 mm. Of these, 1,219 children presented a MUAC<110 and a WH<70% of the median. A total of 11,181 (18.4%) children were admitted with a MUAC between 110 and 119 mm and the majority of children (78.3%, n = 47,566) with a MUAC greater than 120 mm.

**Table 1:** Mean and median weight, height or length, age and WH indices upon admission for all children (n = 68,001).

	Median [IQR]	Mean (SD)
Measure		
Weight (kg)	6.9 [6.0; 7.7]	7.0 (1.4)
Height or length (cm)	72.5 [68.0; 76.5]	73.0 (7.1)
Age (months)	19.0 [13.0; 24.0]	19.5 (8.6)
Indices		
WH Z score (WHO standards)*	−2.95 [−3.4; −2.6]	−3.0 (0.7)
WH % median (NCHS reference)**	76.8 [74.8; 78.2]	76.0 (3.5)

* data missing for 730 children (including presence of edema for 394 children).

** data missing for 674 children.

In terms of type of care, 84.0% (56,582/67,354) of children were admitted as outpatients and 2,157 children (3.2%) were admitted directly into an inpatient center and remained hospitalized until discharge. The remaining 8,615 children (12.8%) received both outpatient and inpatient care. Among all children who were discharged recovered, the median length of stay was 26 days (IQR: 21–35) and the median weight gain was 4.8 g/kg/day (IQR: 3.1–7.0).

Table 2 provides the principal outcomes by malnutrition status according to the NCHS reference. Deaths were concentrated among children receiving inpatient care (n = 348), of which 106 occurred among children who received both inpatient and outpatient care. Of children treated only in outpatient care, 73 died.

**Table 2:** Principal outcome for children admitted in MSF nutritional program, Maradi region, Niger, 2006.

	Moderate acute malnutrition N = 58,126*		Severe acute malnutrition N = 4,684	
	N	(%)	N	(%)
Recovered	55,150	(94.9)	3,717	(79.4)
Died	285	(0.5)	174	(3.7)
Defaulted	2,240	(3.9)	537	(11.5)
Transferred or discharged**	451	(0.8)	256	(5.5)

* Results exclude 3 outpatient centers with unreliable information.

** Children discharged when not responding to treatment: 522.

### Risk Factor Analysis

In total, 27,687 children were included in the risk factors analysis. Table 3 provides a comparison of indicators between surviving children and those that died. Differences between the two were statistically significant for all variables with the exception of Paracheck® results. Sixty-six children (21%) died in the first 48 hours and 162 (51%) by the end of the first week of admission. The median duration from time of admission to time of death was 7 days (IQR: 3–18), with a mean of 14.1 days. It is important to note the very different sex distribution in the sub-sample for the risk factors analysis with WHO standards WH<−3 Z-score (66.4% of males) as compared to the total initial cohort of 68,001 children based on the NCHS reference (44.4% males).

**Table 3:** Indicators of children at admission in MSF nutritional program, Maradi region, Niger, 2006.

Indicator	Description	Surviving (n = 27,315)	Deceased (n = 372)	p*
Gender = male		66.6%	55.5%	0.000
Mean age in months (±s.d)		17.7 (±8.8)	17.6 (±10.6)	0.81
Height (cm)		70.9 (±7.3)	68.5 (±9.0)	0.000
Weight (kg)		6.4 (±1.4)	5.8 (±1.7)	0.000
Mean MUAC (mm) (±s.d)		121.2 (±7.8)	116.6 (±11.7)	0.000
Anorexia	Child refuses to eat RUTF	7.5%	32.3%	0.000
Temperature				
≤38		87.5	79.4	0.000
[38; 39]		8.8	11.4	
>39		3.8	9.3	
Pallor	Clinical assessment	1.1%	7.7%	0.000
Edema	Clinical assessment	1.1%	8.6%	0.000
Nigerian	Nigerian nationality	5.7%	10.8%	0.000
Diarrhea	Recent diarrhea reported by mother	30.3%	35.4%	<0.05
Vomiting	Recent vomiting reported by mother	8.4%	17.2%	0.000
Apathy	Clinical assessment	2.7%	43.9%	0.000
Positive Parachek	Malaria rapid diagnostic test	23.5%	23.0%	0.87

* For continuous variables, the p-value indicates the level of significance from a Student's t-test. For categorical variables, the p-value displayed is for a Chi^2^.

In the first analysis, all the variables tested were retained as possible risk factors in univariate regression, and sex was the only factor not retained in the multivariate model. Coefficients associated to weight and height cannot be interpreted independently, since they are both negatively associated to the risk of death, whereas height is positively associated to this risk at a given weight. In this analysis, they were retained in the optimal ratio of weight/height^1.84^ [6]. Results of the univariate and multivariate analyses are shown in table 4. The following formula was derived from the multivariate model to estimate the individual risk of death:
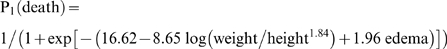



**Table 4:** Results of univariate and multivariate analysis without clinical data.

	Crude OR (univariate analysis)			Adjusted OR (multivariate analysis)		
	Odds ratio	95% CI	p	Odds ratio	95% CI	p
Log (weight)	0.0744	0.0463; 0.0119	0.000	1.75 10^−4^	6.48 10^−5^; 4.73 10^−4^	0.000
Log (height)	0.0635	0.0195; 0.0207	0.000	7.92 10^6^	7.37 10^5^; 8.52 10^7^	0.000
Edema	7.51	5.12; 11.0	0.000	7.10	4.67; 10.8	<0.005
Gender = male	0.638	0.519; 0.785	<0.001			

In the second analysis, which included clinical data, we retained all potential risk factors for death as significant in the univariate analysis, with the sole exception of a positive Paracheck® (Table 5). Risk factors retained in the final model were the weight/height^1.81^ ratio, age below 1 year, anorexia, fever higher than 38.5°C, pallor and edema.

**Table 5:** Results of univariate and multivariate analysis with clinical data.

	Crude OR (univariate analysis)			Adjusted OR (multivariate analysis)		
	Odds ratio	95% CI	p	Odds ratio	95% CI	p
Log (weight)	0.0744	0.0463; 0.0119	0.000	1.94 10^−3^	4.58 10^−4^; 8.20 10^−3^	0.000
Log (height)	0.0635	0.0195; 0.0207	0.000	8.22 10^4^	2.66 10^3^; 2.53 10^6^	0.000
Edema	7.51	5.12; 11.0	0.000	5.23	2.73; 10. 0	0.000
Gender = male	0.638	0.519; 0.785	<0.001			
age<1 year	1.74	1.40; 2.16	0.000	1.42	1.01; 1.99	<0.05
Anorexia	5.89	4.50; 7.72	0.000	1.89	1.35; 2.66	0.000
Fever>38.5°C	2.18	1.60; 2.96	0.000	1.83	1.25; 2.69	<0.002
Pallor	6.44	4.26; 9.72	0.000	2.25	1.25; 4.05	<0.01
Apathy	27.1	21.7; 33.9	0.000	9.71	6.92; 13.6	0.000
Nigerian origin	2.02	1.36; 2.99	0.000			
Diarrhea	1.26	1.00; 1.58	<0.05			
Vomiting	2.13	1.60; 2.83	0.000			
Positive Parachek	1.02	0.794; 1.31	0.87			

Regardless of the variable selection method, after controlling for potential confounders and interactions and stratified models no different predictors of death emerged. This multivariate analysis was used to propose a second formula to estimate the individual risk of death when perfunctory clinical data are available:
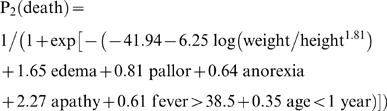



## Discussion

As the release of WHO growth standards changed the definition of severe malnutrition, which now includes many children formerly identified as moderately malnourished with the NCHS reference, studying this new category of children is crucial. Within the 32,933 children identified in our sample as severely malnourished according to the WHO standards (see figure 2), 28,093 (85.3%) were not classified as such by the former NCHS reference (i.e. they were “WHO severes” with WH<−3 WHO standards Z-score, but “NCHS moderates” with WH≥70% of the NCHS median). Feeding programs aimed at severe malnutrition are thus likely to include larger numbers of children with better overall nutritional status in the near future. Nutritional actors will need to think about appropriate strategies to manage these “new severes”.

The first model reported here, without clinical observation and based on basic data recorded at admission in most nutritional programs, gives a result close to the model proposed by Prudhon *et al* (who used the NCHS reference). The risk factors for death identified in the multivariate analysis including clinical data hint either towards concomitant infection (i.e., fever) or known features of malnutrition (e.g. low weight-for-height). In light of the magnitude of this database, the absence of association between death and sex, diarrhea, vomiting and Paracheck® positivity can be regarded as a robust finding, which is also consistent with previously published work [10], [16] based on the NCHS growth references.

A potentially innovative aspect of our results is the good performance of the “appetite test” for the assessment of anorexia. Plumpy'nut® has shown to be popular with malnourished children in previous trials [17], [18]. This simple test, easy to implement in therapeutic feeding programs, still requires evaluation in an appropriately designed study comparing against conventional criteria for anorexia.. Quantity of RUTF intake, time allowed for eating and method of observation by caregivers should be standardized (for example quantity of RUTF expected to be eaten per 10 minutes for a given age).

We did not find a positive Paracheck® to be associated with a higher risk of death. This is likely because unlike other potential concomitant infections, malaria benefits from a reliable rapid diagnostic test. This allowed for rapid and effective treatment (artesunate+amodiaquine). We also did not find children originating from Nigeria to be at higher risk of death. We may have suspected Nigerian children to be at higher risk as they traveled long distances to seek treatment and may have been in worse condition.

This analysis highlights the importance of identifying simple and robust indicators for determining whether a child should be placed in outpatient or inpatient care, a key strategy for rational management of community-based programs. In such context, both formula derived from these models may help to build a “risk score” for early triage and rapid referral to inpatient care. One option could be to establish differential management for different types of “new severes” according to anthropometrical and clinical criteria. This could imply up-stream screening by less medically qualified staff, based on precise criteria, associated with a greater risk of death. As a monitoring tool, these formula could also be helpful to examine mortality trends (e.g. after a modification of a treatment protocol).

We note the limitations of using program monitoring data to investigate epidemiological questions in nutrition such as risk factors for death. Although a large number of additional indicators upon admission were collected, variations between clinicians in how these data were recorded prevented their analysis. Breastfeeding practice is a notable example. This is of course important to consider, but the information recorded was not reliable enough to be included in our analyses. Some clinical signs may be due not to acute malnutrition but to a concomitant pathology, (for example apathy can be linked to severe wasting or to another cause like infection or dehydration, as well as pallor can be due to nutritional micronutrient deficiency or to malaria anemia without any malnutrition component). They are moreover “subjective” signs, and their finding and reporting may be clinician-dependant. Detailed prospective studies of co-morbidities and clinical signs at admission are required to improve further the treatment of malnutrition. We limited our analysis to examining indicators where we felt the data collection and the clinical criteria were sufficiently strong over this large database. Future analysis should include quality documentation of metabolic and clinical status, and response to therapy, particularly in the first few days following admission.

Fatality was low in the MSF program, far below the 5% case fatality rate often expressed as a threshold for acceptability in hospitals [19]. However, case fatality is likely to be underestimated as some children who were lost to follow up from the outpatient program might have died at home. This is an area for additional improvement with more rigorous tracing of defaulters. In accordance with previous findings [20], most deaths occurred within 7 days of admission. This highlights the importance of ensuring adequate access to effective treatment, community awareness activities encouraging parents to bring their children early for treatment and strategies to detect children at earlier stages of malnutrition. The median age of children admitted to the program was approximately 18 months, with a median weight of approximately 6 kg. Detecting and treating these children earlier is a clear priority. These early deaths also reinforce the importance of studies examining the etiology of death among malnourished children, in particular concomitant infection(s).

### Conclusion

As the transition from the NCHS growth reference to the WHO standards will increase the number of children classified as severely malnourished, up-stream screening based on anthropometrical and clinical signs may be crucial in adapting the management strategy. Because the triage must rely on basic-level health workers, criteria developed should be easy, precise and clear, based on robust evidence. Our findings provide risk factors for mortality– weight, height, edema, pallor, anorexia, apathy and fever – that can be proposed for building criteria for the orientation of children towards either hospitalization or ambulatory care
